# Supplementary material for: Prescription of oral antidiabetics in Mexico. A cross-sectional study
Source: PLoS One. 2023 Jul 27;18(7):e0288699. doi: 10.1371/journal.pone.0288699 (PMC10373993; doi:10.1371/journal.pone.0288699)
Supplement: Attachment — Submitted filename: Response to Reviewers.docx [file pone.0288699.s001.docx]

Academic Editor

PLOS ONE

Dear Editor,

Thank you for giving us the opportunity to resubmit the manuscript. We have revised the document according to the comments and suggestions that have been made. Corrections have been introduced as indicated and a marked-up copy of the manuscript that highlights changes made to the original version has been built-up. Changes appear in red.

We do thank you for your work. No doubt, the comments and suggestions provided have contributed to noticeably improve the manuscript.

Sincerely yours,

Juan Molina

Corresponding author

**Points raised by the academic editor and reviewers**

**Editor**

1. *“Please ensure that your manuscript meets PLOS ONE's style requirements […]”.* We have checked the manuscript to meet the journal´s requirements.
2. *“We noticed you have some minor occurrence of overlapping text with the following previous publication(s), which needs to be addressed: Prescription patterns of antihypertensives in a community health centre in Mexico City: a drug utilization study -* [*https://doi.org/10.1111/fcp.12179*](https://urldefense.com/v3/__https:/doi.org/10.1111/fcp.12179__;!!D9dNQwwGXtA!RP2yHal8foj0PUyrondTH3jY6CJdg7ll3CsvAAN-dbAziVi0X_PP7TmCULBWE3eeqeecxoXgjdzTnyV0K34$)*”.* There is in fact an overlapping text: “For the purpose, a retrospective cross-sectional drug utilization study has been conducted” and “Most of the results are expressed in frequencies and percentages”. They have previously appeared in the Methods section of a previous article by our group; they have a general character and do not affect the originality of the present article. There is no duplicate text outside the methods section.
3. *“Please provide additional details regarding participant consent. In the ethics statement in the Methods and online submission information, please ensure that you have specified (1) whether consent was informed and (2) what type you obtained (for instance, written or verbal, and if verbal, how it was documented and witnessed). If your study included minors, state whether you obtained consent from parents or guardians. If the need for consent was waived by the ethics committee, please include this information.*

*If you are reporting a retrospective study of medical records or archived samples, please ensure that you have discussed whether all data were fully anonymized before you accessed them and/or whether the IRB or ethics committee waived the requirement for informed consent. If patients provided informed written consent to have data from their medical records used in research, please include this information.”* According to your suggestion, we have re-written the “ethics statement” in the Methods section as follows, “This project was filed as self-determined activities not regulated as human participant research with the Hospital Regional de Alta Especialidad de Ixtapaluca (HRAEI) institutional Ethics Committee; this institutional review board which approved the study is an appropriately constituted group that has been formally designated to review and monitor biomedical research involving human subjects, and it is also formally registered in the Mexican official list for investigational ethics committees. All data were fully anonymized before we accessed them.

*“In your Data Availability statement, you have not specified where the minimal data set underlying the results described in your manuscript can be found […]*

We have made data available and we have introduced new information: The data are available at [https://data.mendeley.com/datasets/mjjcvhdxk6/1](https://urldefense.com/v3/__https:/data.mendeley.com/datasets/mjjcvhdxk6/1__;!!D9dNQwwGXtA!UjmvUeRqdrzxE_c82V1NpUsB9JMDPue81sxPPYNTFm7KJGp5pD9pb1E7r2XrWWYTNA2CJoJQniGDR-k4nkA$)

**Reviewer 1**

1. Introduction section. *It is recommended [to] “summarize the existing literature on this topic, identify the gap in knowledge and mention how their study aims to fill that gap in knowledge in the introduction section of the manuscript”.* There is current general agreement about the shortage of studies addressing the patterns of use of antidiabetics and its correctness in Mexico (Corona-Rojo JA et al. Potential prescription patterns and errors in elderly adult patients attending public primary health care centers in Mexico City. Clin Interv Aging. 2009;4: 343-50. https://doi.org/10.2147/cia.s5198). In the submitted manuscript, we identified and referenced two published studies upon “inappropriate prescription” (Mino-León D et al. Potentially inappropriate prescribing to older adults in ambulatory care: prevalence and associated patient conditions. Eur Geriatr Med. 2019;10: 639–647; Zavala-González MA et al. Utilización de hipoglucemiantes orales en una unidad médica 392 familiar de Comalcalco, Tabasco, México, 2013. Rev. Mex. Cien. Farm. 2014;45: 81-85); they were quoted in the Discussion section of the manuscript. As suggested, a new search has been conducted identifying a new study that we have accordingly incorporated to the manuscript (Cardoza-Contreras D et al. Inappropriate Drug Prescription in the Elderly in Tijuana. J Fam Med, 2019, vol. 6, no 2, p. 1161). To summarize the existing literature, we have quoted the three studies and briefly described them in the Introduction section regardless their quotation in the Discussion section.
2. Methods section *(page 5, lines 107-110). It is stated that, “though the authors have mentioned what information was retrieved from the records, it would improve the manuscript, if the authors briefly mention who retrieved the information, their respective qualifications”*. Osiel Gallardo-Mora (OG-M), one of the signing authors of this manuscript, retrieved the information from the records. We have accordingly introduced this information in the revised manuscript as follows: “All relevant information was retrieved, with a common template, by one of the signing authors (OG-M); he is a qualified medical doctor”.
3. Methods section *(page 5, lines 118-124). “…though the authors have mentioned that the study did not require informed consent, and that the study meets the requirements of current Mexican law regarding health research, it would add to the quality of the manuscript if the authors mention a few lines regarding the institutional review board (IRB) approval as well”.* The Hospital Regional de Alta Especialidad de Ixtapaluca (HRAEI) IRB is an appropriately constituted group that has been formally designated to review and monitor biomedical research involving human subjects; in accordance, it is formally registered (number 127) in the Mexican official list for investigational ethics committees (<https://www.gob.mx/cms/uploads/attachment/file/442503/Registros_CEI_25022019.pdf>). Since another remark upon this point has been posed by the Editor, further information has been introduced in the corresponding section (see responses to questions posed by the editor).
4. Discussion section. *It is suggested that “clinical implications of their findings, how their findings expand on the existing literature, the future research implications, in the discussion section of the manuscript” [should be mentioned].* Regarding clinical implications, it is said in the current manuscript that “A clinical practice for managing T2DM in accordance with current guidelines may avoid inappropriate prescription and improve attention; it also may contribute to a better glycemia control in these patients”. Since “nutritional anamnesis” appears to avoid inappropriate prescription, the following statement has been added: “More specifically, paying attention to nutritional anamnesis might be of particular interest according to what was observed in the present study”.
5. General. *“…thoroughly proofreading…”.* See point 1 posed by Reviewer 1.

**Reviewer 2**

1. General. *It is said that “the article needs a re-write with appropriate grammatical construction and scientific language”.* This is coincidental with that suggested by Reviewer 1. The whole manuscript has been thoroughly proofread to rectify the typos and grammatical errors and, when applied, re-written with scientific language.
2. Results section. *“Line 147-150 talks about the glycemic values in the control population. 36.5% had within recommended range and then 85% had uncontrolled DM”.* It is in fact an error, we do apologize. The first percentage (36.5%), coincidental with figures in Table 1 and in the Abstract, is the correct one; the last one (85%) has been deleted.
3. Discussion section. *“The authors need to mention strategies of how to improve diabetes control”.* This requirement is in part like that suggested by Reviewer 1. Diabetes control is a target difficult to achieve. In a recently published cross-sectional analysis of data from adults with diabetes in the United States participating in the National Health and Nutrition Examination Survey to assess national trends in diabetes treatment and risk-factor control from 1999 through 2018, the percentage of adult participants with diabetes, in whom glycemic control (glycated hemoglobin level, <7%) was achieved, declined from 57.4% (2007-2010; n=1481) to 50.5% (2015-2018; n=1718). [Fang M et al. Trends in Diabetes Treatment and Control in U.S. Adults, 1999-2018. N Engl J Med. 2021; 384 (23):2219-2228]. Diabetes control implies different stakeholders: health authorities, health professionals and patients themselves; scientific work somehow may help. We have re-written this point as follows, “A clinical practice for managing T2DM in line with current guidelines may avoid inappropriate prescription and improve attention; it also may contribute to a better glycemia control in these patients. More specifically, paying attention to nutritional anamnesis might be of particular interest according to what was observed in the present study”.

**Additional minor amendments.** Other amendments have been performed. Some errors not affecting the overall results have been corrected and appropriately indicated in red in the Results section. Similarly, an update in the number of deaths attributed to diabetes in Mexico has been introduced in the Discussion section; the corresponding source has accordingly been referenced (INEGI. Estadísticas de las defunciones registradas en México, 2021).

**All changes have been introduced, in red, in the 'Revised Manuscript with Track Changes.'**
