# Supplementary material for: Prescription of oral antidiabetics in Mexico. A cross-sectional study
Source: PLoS One. 2023 Jul 27;18(7):e0288699. doi: 10.1371/journal.pone.0288699 (PMC10373993; doi:10.1371/journal.pone.0288699)
Supplement: Attachment — Submitted filename: Response to Reviewers.docx [file pone.0288699.s002.docx]

Academic Editor

PLOS ONE

Dear Editor,

Thank you for giving us the opportunity to resubmit the manuscript. We have revised the document according to the comments and suggestions that have been made. Corrections have been introduced as indicated and a marked-up copy of the manuscript that highlights changes made to the original version has been built-up. Changes appear in red.

We do thank you for your work. No doubt, the comments and suggestions provided have contributed to noticeably improve the manuscript.

Sincerely yours,

Juan Molina

Corresponding author

**Point raised.** *Page 6, methods section, line 134: while the authors have acknowledged that all data was anonymized, it is recommended that they also mention whether the ethics committee granted a waiver for informed consent.*

**Answer.** Given the characteristics of the study based on anonymized retrospective data, the Committee, bearing in mind Mexican legislation on research with humans’ beings [1] and on data protection [2], did not consider the explicit exemption of informed consent necessary. Nevertheless, the Hospital Ethics Committee authorized and oversaw data anonymization.

[1] REGULATION OF THE GENERAL HEALTH LAW ON HEALTH RESEARCH Last reform published DOF 02-04-2014. <https://www.diputados.gob.mx/LeyesBiblio/regley/Reg_LGS_MIS.pdf>

(Art. 17) Research without risk: These are studies that use retrospective documentary research techniques and methods and those in which no intervention or intentional modification is made in the physiological, psychological, and social variables of the individuals participating in the study, among which are considered: questionnaires, interviews, review of clinical records and others, in which sensitive aspects of their behavior are not identified or treated.

[2] FEDERAL LAW ON PROTECTION OF PERSONAL DATA HELD BY PRIVATE PARTIES New Law published in the Official Gazette of the Federation on July 5, 2010 <https://www.diputados.gob.mx/LeyesBiblio/pdf/LFPDPPP.pdf>

(Art. 10) Consent will not be necessary for the processing of personal data when: I. It is provided for in a Law; II. The data are contained in publicly accessible sources; III. Personal data are subject to a prior dissociation procedure. (Dissociation: The procedure by which personal data cannot be associated with the owner or allow, due to its structure, content or degree of disaggregation, the identification of the same).

Thus, an additional statement in the corresponding Methods section has been introduced, “Given the characteristics of the study based on anonymized retrospective data, the Committee, bearing in mind Mexican legislation on research with human beings and on data protection, did not consider the explicit exemption of informed consent necessary. Nevertheless, the Hospital Ethics Committee authorized and oversaw data anonymization”.

**All changes have been introduced, in red, in the 'Revised Manuscript with Track Changes.'**
